# Reassortant Avian Influenza A(H5N1) Viruses with H9N2-PB1 Gene in Poultry, Bangladesh

**DOI:** 10.3201/eid1910.130534

**Published:** 2013-10

**Authors:** Isabella Monne, Mat Yamage, Gwenaëlle Dauphin, Filip Claes, Garba Ahmed, Mohammed Giasuddin, Annalisa Salviato, Silvia Ormelli, Francesco Bonfante, Alessia Schivo, Giovanni Cattoli

**Affiliations:** Istituto Zooprofilattico Sperimentale delle Venezie, Padova, Italy (I. Monne, A. Salviato, S. Ormelli, F. Bonfante, A. Schivo, G. Cattoli);; Food and Agriculture Organization of the United Nations Emergency Center for Transboundary Animal Diseases, Dhaka, Bangladesh (M. Yamage, G. Ahmed);; Food and Agriculture Organization of the United Nations Animal Health Service, Rome, Italy (G. Dauphin, F. Claes);; Bangladesh Livestock Research Institute National Reference Laboratory for Avian Influenza, Dhaka (M. Giasuddin)

**Keywords:** Highly pathogenic avian influenza virus H5N1, Bangladesh, phylogenetic analysis, reassortment, viruses, influenza, influenza (H5N1), avian influenza, HPAI, H9N2-PB1, H9N2, reassortant, avian influenza A(H5N1), genes, zoonoses

## Abstract

Bangladesh has reported a high number of outbreaks of highly pathogenic avian influenza (HPAI) (H5N1) in poultry. We identified a natural reassortant HPAI (H5N1) virus containing a H9N2-PB1 gene in poultry in Bangladesh. Our findings highlight the risks for prolonged co-circulation of avian influenza viruses and the need to monitor their evolution.

Bangladesh has one of the highest reported number of outbreaks of highly pathogenic avian influenza (HPAI) (H5N1) in poultry (*1*). As of May 26, 2013, a total of 548 outbreaks of HPAI (H5N1) have been reported (*1*); these outbreaks have resulted in serious economic repercussions in the poultry sector in this country. Furthermore, 7 cases of human infection with HPAI (H5N1) were confirmed, most recently a fatal case in a 2-year-old child in April 2013 (*2*).

In addition to the HPAI (H5N1) virus, the H9N2 subtype is widely circulating in poultry in Bangladesh, which raises concerns about the possible implications of the extensive co-circulation of these viruses (*3*,*4*). Their coexistence in the same susceptible population is likely to generate appropriate conditions for the emergence of novel reassortant variants, with unknown epizootic and zoonotic potential. We characterized the complete genome of 18 HPAI (H5N1) virus strains from Bangladesh and report the identification and characterization of a novel natural reassortant HPAI (H5N1) virus that contains an H9N2-PB1 gene in poultry.

## The Study

A total of 18 tracheal and 1 cloacal samples were collected from chickens in 14 layer farms (15 samples) and 2 live-bird markets (4 samples) in 13 districts of Bangladesh during December 2011–April 2012. The samples were submitted by the Department of Livestock Services Dhaka to the World Organisation for Animal Health/United Nations Food and Agriculture Organization Reference laboratory for Avian Influenza in Italy for confirmatory diagnosis and genetic analysis. All samples tested positive for the H5N1 subtype by real-time reverse transcription PCR (rRT-PCR) (*5*) and virus isolation (http://www.oie.int/fileadmin/Home/eng/Health_standards/tahm/2.03.04_AI.pdf). Because the H7 subtype is also notifiable and the H9N2 subtype circulates in the poultry population in Bangladesh (*3*), samples were tested for these subtypes as well; results were negative. Epidemiologic information for these viruses is provided in the Table.

**Table:** Avian influenza A(H5N1) virus strains analyzed in study of reassortant viruses in poultry, Bangladesh, December 2011–April 2012

Strain	Division, district, city	GPS coordinates, decimal degrees, N, E	Date of sample collection	Type of production
A/chicken/Bangladesh/Khulna/12VIR-7140–1/2011	Khulna, Bagerhat, Sonatala	22.477883, 89.652883	Dec 19	Layer
A/chicken/Bangladesh/Khulna/12VIR-7140–2/2012	Khulna, Meherpur	23.775000, 88.6417	Jan 2	Layer
A/chicken/Bangladesh/Dhaka/12VIR-7140–3/2012	Dhaka, Demra	23.707533, 0.471667	Jan 8	Layer (hatchery)
A/chicken/Bangladesh/Dhaka/12VIR-7140–4/2012	Dhaka, Manikgahj, Shibalaya	23.828967, 89.7854	Jan 10	Layer (hatchery)
A/chicken/Bangladesh/Dhaka12VIR-7140–5/2012	Dhaka, Narayanganj, Araihajar	23.78395, 90.6517	Jan 21	Layer (hatchery)
A/chicken/Bangladesh/Dhaka12VIR-7140–6/2012	Dhaka, Tangail	24.684433, 89.95545	Feb 14	Layer (hatchery)
A/chicken/Bangladesh/Rajshahi/12VIR-7140–7/2012	Rajshahi, Sirajganj, Tarash	24.363633, 89.361617	Jan 19	Layer (hatchery)
A/chicken/Bangladesh/Chittagong/12VIR-7140–8/2012	Chittagong, Feni	22.9294, 91.300933	Feb 14	Layer (hatchery)
A/chicken/Bangladesh/Dhaka/12VIR-7140–9/2012	Dhaka, Gazipur	23.89306, 90.330319	Feb 16	Layer (hatchery)
A/chicken/Bangladesh/Khulna/12VIR-7140–10/2012	Khulna, Khulna	22.708517, 89.353517	Mar 5	Layer (hatchery)
A/chicken/Bangladesh/Dhaka/12VIR-7140–11/2012	Dhaka, Dhaka, Dohar	23.6091, 90.13205	Mar 18	Layer (hatchery)
A/chicken/Bangladesh/Khulna/12VIR-7140–12/2012	Khulna, Jessore	23.182367, 89.190183	Mar 27	Layer (hatchery)
A/chicken/Bangladesh/Rangpur/12VIR-7140–13/2012	Rangpur, Nilphamari	25.91686, 88.98789	Apr 3	Layer
A/chicken/Bangladesh/Rangpur/12VIR-7140–14/2012	Rangpur, Nilphamari	25.91686, 88.98789	Apr 3	Layer
A/chicken/Bangladesh/Dhaka/12VIR-7140–15/2012	Dhaka, Rajbari, Pangsha	23.736233, 89.54385	Apr 5	Layer (hatchery)
A/chicken/Bangladesh/Dhaka/12VIR-7140–16/201	Dhaka (Kaptan Bazar), Dhaka, Dhaka	23.716765, 90.400245	Mar 13	Live-bird market
A/chicken/Bangladesh/Dhaka/12VIR-7140–17/2012	Dhaka (Anando Bazar), Rajbari, Dhaka	23.716783, 90.400053	Mar 21	Live-bird market
A/chicken/Bangladesh/Dhaka/12VIR-7140–18/2012	Dhaka (Tejgaon Bazar), Dhaka, Dhaka	23.750155, 90.383516	Mar 21	Live-bird market
A/chicken/Bangladesh/Dhaka/12VIR-7140–19/2012	Dhaka (Kaptan Bazar), Dhaka, Dhaka	23.716765, 90.400245	Mar 25	Live-bird market

The complete genome of 18 HPAI (H5N1) isolates and the hemagglutinin (HA) gene of 1 virus (A/chicken/Bangladesh/12VIR-7140-9/2012) were successfully sequenced, as described (*6*). The nucleotide sequences obtained in this study have been submitted to GenBank (accession nos. KC616462–KC616606). Maximum-likelihood (ML) trees were estimated for all 8 gene segments (HA, neuraminidase [NA], nucleoprotein [NP], basic polymerase protein [PB] 1 and 2, polymerase [PA], matrix [M], and nonstructural [NS]) by using the best-fit general time reversible + I + Γ4 model of nucleotide substitution in PhyML (*7*). A bootstrap resampling process (1,000 replications) using the neighbor-joining method was used to assess the robustness of individual nodes of the phylogeny, incorporating the ML substitution model defined above.

**Figure 1:**
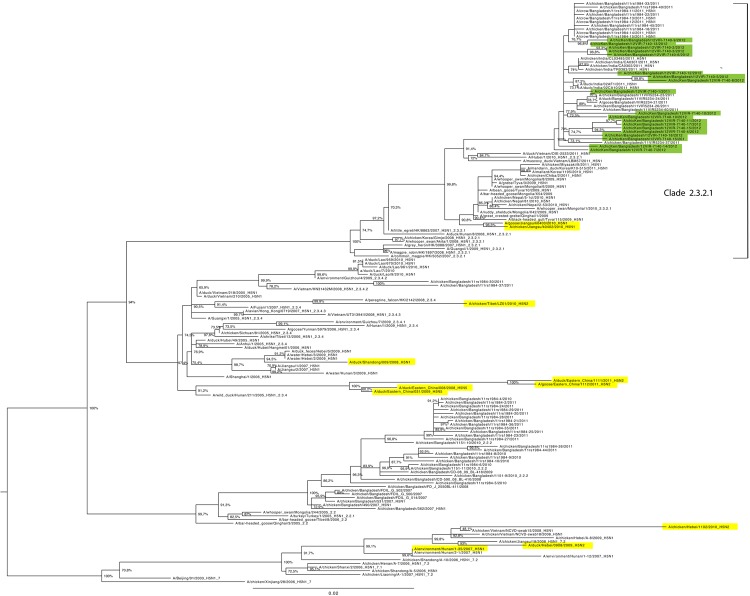
Maximum-likelihood phylogenetic tree for the hemagglutinin gene segment of avian influenza (H5N1) viruses from Bangladesh compared with other viruses. Green shading indicates viruses from Bangladesh sequenced and characterized in this study; yellow shading indicates previously described subtype H5N1/H9N2 reassortant influenza viruses (*8*,*9*) or those from GenBank. Numbers at the nodes represent bootstrap values. Scale bar indicates nucleotide substitutions per site.

Analysis of the topologic differences between the 8 ML phylogenetic trees revealed that an intersubtype reassortment event took place and caused replacement of the PB1 gene of 2 of the HPAI (H5N1) viruses from Bangladesh (A/chicken/Bangladesh/12VIR-7140-7/2012 and A/chicken/Bangladesh/12VIR-7140-16/2012) with the PB1 gene of an H9N2 subtype virus. In the HA gene, all the viruses from Bangladesh analyzed fell within clade 2.3.2.1 (Figure 1) and had high similarity (98.2%–99.7%) to viruses from Bangladesh and India identified in 2011 (*10*). Phylogenetic analysis of the NA, NP, PB2, PA, M, and NS genes (Technical Appendix) confirmed the clustering observed for the HA gene; all 18 fully sequenced viruses grouped together and with viruses from Bangladesh and India identified in 2011.

A close examination of the PB1 tree topology revealed the existence of a diversified origin of this internal gene for the viruses analyzed. In particular, the PB1 gene of 16 of the 18 viruses we characterized clustered as described for the other 7 genome segments, whereas phylogeny of A/chicken/Bangladesh/12VIR-7140-7/2012 and A/chicken/Bangladesh/12VIR-7140-16/2012 revealed that their PB1 gene derived from H9N2 subtype viruses. In particular, the PB1 gene of these 2 HPAI (H5N1) strains belongs to a cluster composed by subtype H9N2 viruses from Bangladesh and India identified during 2008–2011 and had the highest similarity (96.9%–97.1%) with the Indian strain A/chicken/Tripura/105131/2008. Genetic identity between A/chicken/Bangladesh/12VIR-7140-7/2012 and A/chicken/Bangladesh/12VIR-7140-16/2012 ranged from 98.1% for the PA gene to 99.3% for the NP gene; the 2 viruses can be distinguished from each other by a total of 37 aa differences found in the 13 viral proteins, with the PA gene possessing most (16) of these substitutions. Furthermore, distinct epidemiologic origins characterize these viruses; the A/chicken/Bangladesh/12VIR-7140-7/2012 virus was identified in a chicken farm located in Sirajganj district in January 2012, whereas the A/chicken/Bangladesh/12VIR-7140-16/2012 strain was detected 2 months later (March 2012) from organs collected in Kaptan Bazar in Dhaka (Table), which is 126 km from Sirajganj district. By evaluating these findings, we can theorize that a single reassortant event occurred and then the viruses evolved independently or that 2 independent reassortant events took place.

**Figure 2:**
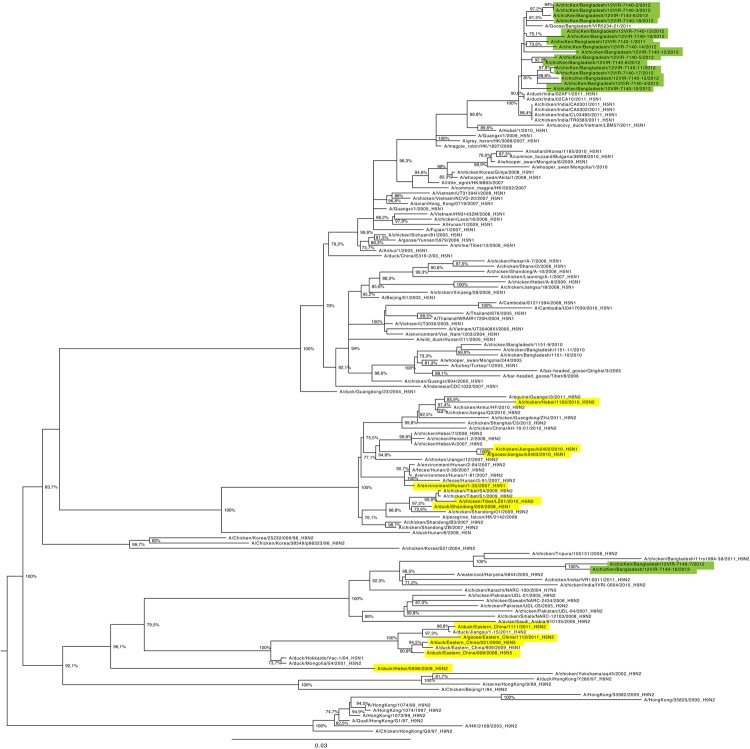
Maximum-likelihood phylogenetic tree for the basic polymerase 1 gene segment of avian influenza (H5N1) viruses from Bangladesh compared with other viruses. Green shading indicates viruses from Bangladesh sequenced and characterized in this study; yellow shading indicates previously described subtype H5N1/H9N2 reassortant influenza viruses (*8*,*9*) or those from GenBank. Numbers at the nodes represent bootstrap values. Scale bar indicates nucleotide substitutions per site.

The genetic characterization of the 2 natural reassortant viruses we identified was remarkably different from that of previously identified reassortant viruses possessing subtype H9N2 genes in an HPAI (H5N1) backbone virus identified in Asia (*8*,*9*). Our results imply that these natural reassortments occurred independently. Indeed, the phylogenic trees for the HA and PB1 genes (Figures 1, 2) demonstrate that the 2 viruses from Bangladesh can be distinguished from the H5N1/H9N2 reassortant strains identified in Tibet and China.

The pathogenicity of 3 subtype H5N1 viruses (the 2 H5N1/H9N2 7:1 reassortants and the H5N1 A/chicken/Bangladesh/12VIR-7140–12/2012 isolate) was determined by intravenous pathogenicity index in specific pathogen–free chickens. This value was 3.0 for all the viruses tested, confirming the isolates can be considered highly pathogenic, according to the World Organisation for Animal Health definition (www.oie.int/fileadmin/Home/eng/Health_standards/tahm/2.03.04_AI.pdf).

## Conclusions

Although reassortment represents a major mechanism behind the emergence and evolution of the HPAI (H5N1) in Asia (*11*,*12*), data suggest that, in the past decade, these viruses have only sporadically provided the backbone for generation of novel intersubtype reassortants (*8*,*9*,*13*,*14*). This is surprising considering the prolonged and extensive co-circulation of subtype H5N1 and H9N2 viruses in poultry in Asia; it is unclear whether the limited evidence is because of insufficient surveillance or the low capacity of subtype H5N1 viruses to undergo intersubtype reassortment. We have documented the emergence and circulation of a novel natural H5N1/H9N2 7:1 reassortant strain in Bangladesh in 2012. This finding shows that the HPAI (H5N1) viruses circulating in this area are continuously evolving. Although it is difficult to ascertain where and when the reassortment event occurred, the identification of genetic clustering of the strains analyzed here with subtype H5N1 and H9N2 viruses identified in India confirms intense cross-border transmission between these regions, which has resulted in a favorable epidemiologic system for influenza viruses evolution (*15*).

The emergence of these natural H5N1/H9N2 reassortant influenza viruses suggests that co-infections with viruses of different subtypes have presumably occurred in poultry, most likely a result of the persistent co-circulation of these viruses along with poor biosecurity measures. This possibility underlines the importance of providing poultry farmers and small-holder poultry producers with educational programs about appropriate control measures for avian influenza. Furthermore, constant efforts must be undertaken to continue monitoring the evolution of influenza A(H5N1) viruses in Bangladesh and bordering countries to estimate the spread of this novel variant and to trace its origin and evolution. There is evidence that the polymerase subunits have a key role in virus replication efficiency and cross-species transmission; therefore, pathogenicity and transmission studies in poultry and mammal models are essential to evaluate the potential animal and public health threat posed by these novel H5N1/H9N2 reassortant influenza viruses.

Technical Appendix Maximum-likelihood phylogenetic trees for the neuraminidase, basic polymerase 2, polymerase, nonstructural, and matrix gene segments of avian influenza (H5N1) viruses from Bangladesh compared with other viruses.
