# Supplementary material for: Reassortant Avian Influenza A(H5N1) Viruses with H9N2-PB1 Gene in Poultry, Bangladesh
Source: Emerg Infect Dis. 2013 Oct;19(10):1630–4. doi: 10.3201/eid1910.130534 (PMC3811991; doi:10.3201/eid1910.130534)
Supplement: Technical Appendix — Maximum-likelihood phylogenetic trees for the neuraminidase, basic polymerase 2, polymerase, nonstructural, and matrix gene segments of avian influenza (H5N1) viruses from Bangladesh compared with other viruses. [file 13-0534-Techapp-s1.pdf]

Article DOI: http://dx.doi.org/10.3201/eid1910.130534

Reassortant Avian Influenza A(H5N1) Viruses with H9N2-PB1 Gene in Poultry, Bangladesh

Technical Appendix

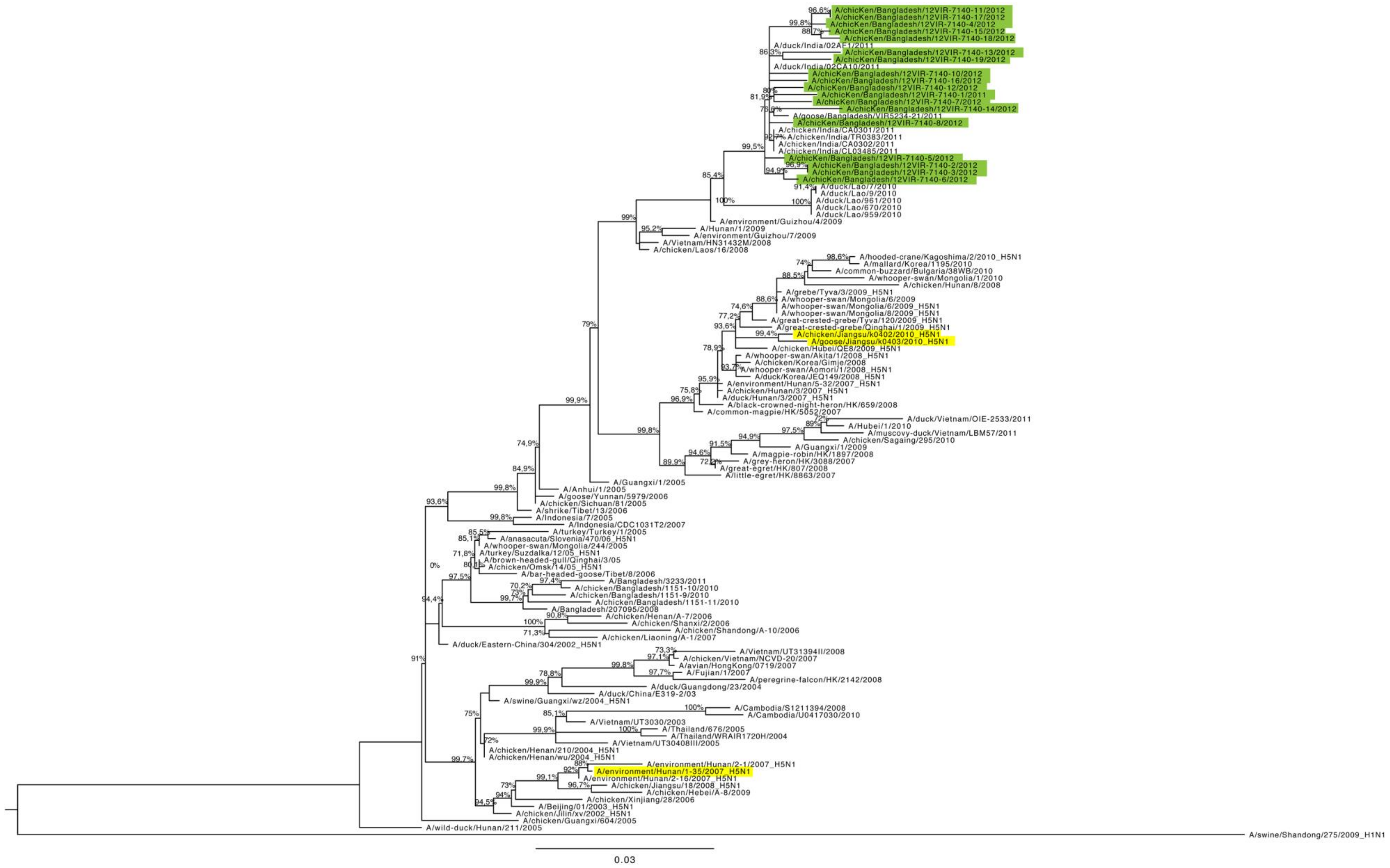

Technical Appendix Figure 1. Maximum-likelihood phylogenetic tree for the neuraminidase gene segment of avian influenza (H5N1) viruses from Bangladesh compared with other viruses. Green shading indicates viruses from Bangladesh sequenced and characterized in this study; yellow shading indicates previously described subtype H5N1/H9N2 reassortant influenza viruses (*1*,*2*) or those from GenBank. Numbers at the nodes represent bootstrap values.

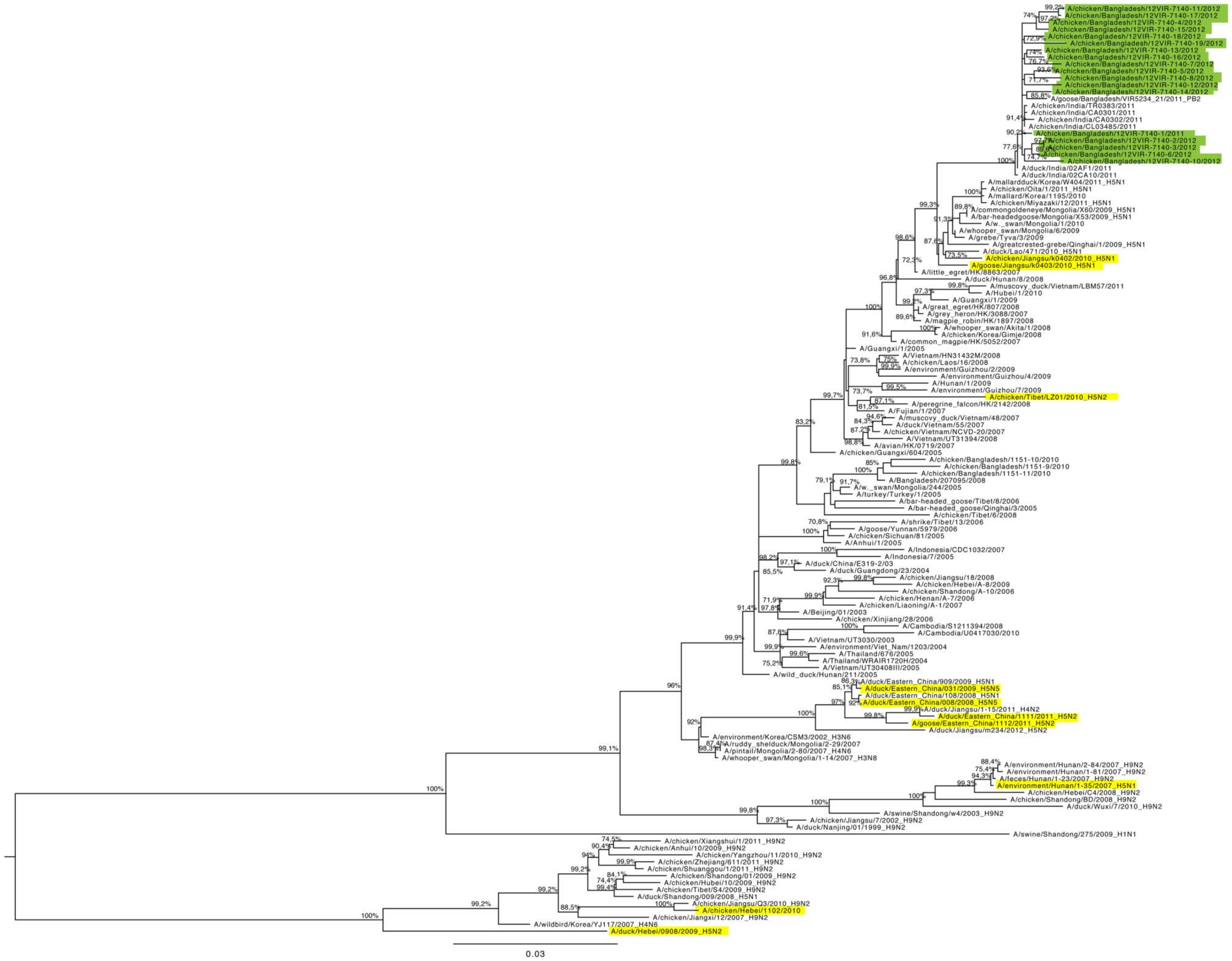

Technical Appendix Figure 2. Maximum-likelihood phylogenetic tree for the basic polymerase 2 gene segment of avian influenza (H5N1) viruses from Bangladesh compared with other viruses. Green shading indicates viruses from Bangladesh sequenced and characterized in this study; yellow shading indicates previously described subtype H5N1/H9N2 reassortant influenza viruses (*1*,*2*) or those from GenBank. Numbers at the nodes represent bootstrap values.

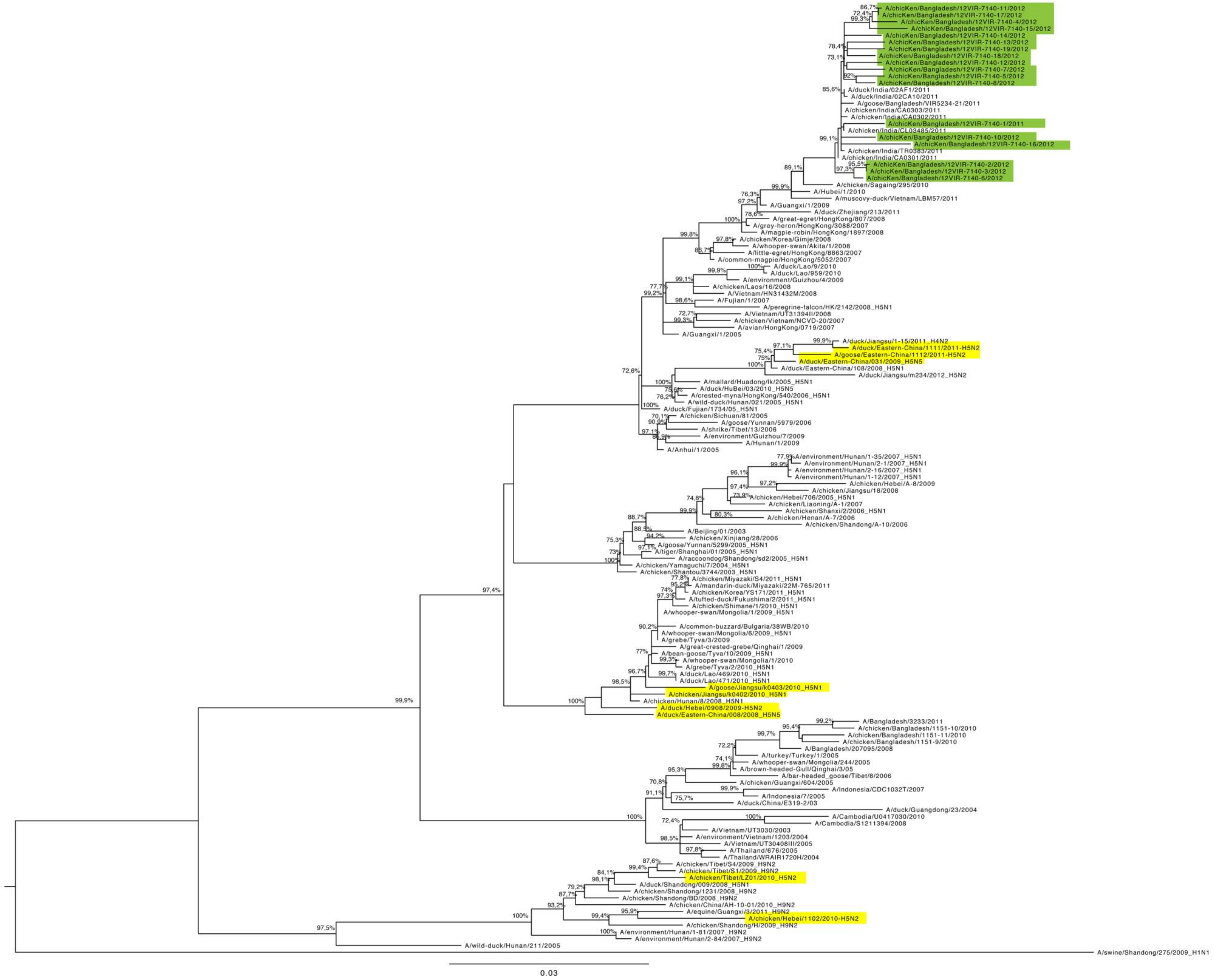

Technical Appendix Figure 3. Maximum-likelihood phylogenetic tree for the polymerase gene segment of avian influenza (H5N1) viruses from Bangladesh compared with other viruses. Green shading indicates viruses from Bangladesh sequenced and characterized in this study; yellow shading indicates previously described subtype H5N1/H9N2 reassortant influenza viruses (*1*,*2*) or those from GenBank. Numbers at the nodes represent bootstrap values.

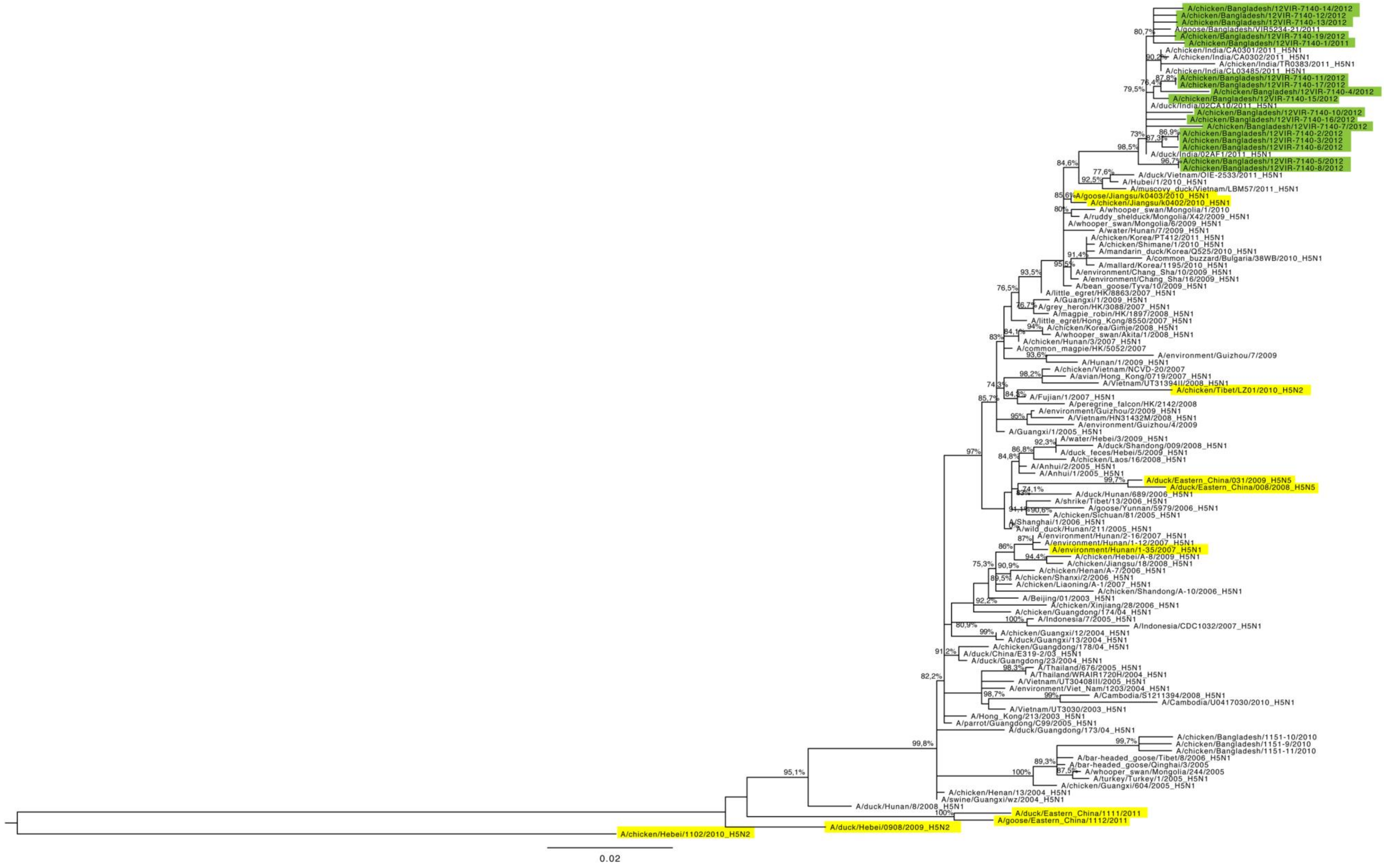

Technical Appendix Figure 4. Maximum-likelihood phylogenetic tree for the nonstructural gene segment of avian influenza (H5N1) viruses from Bangladesh compared with other viruses. Green shading indicates viruses from Bangladesh sequenced and characterized in this study; yellow shading indicates previously described subtype H5N1/H9N2 reassortant influenza viruses (*1*,*2*) or those from GenBank. Numbers at the nodes represent bootstrap values.

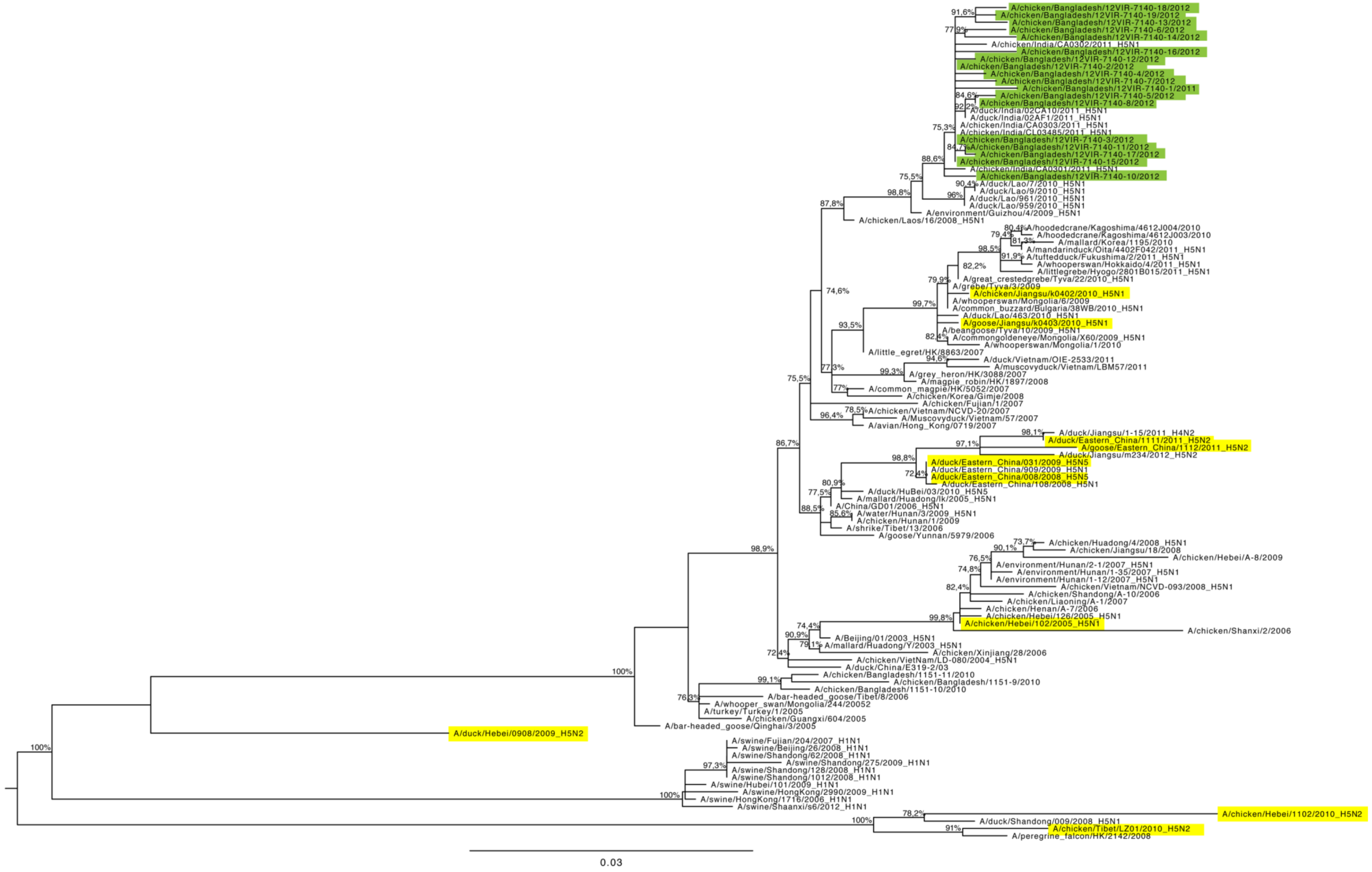

Technical Appendix Figure 5. Maximum-likelihood phylogenetic tree for the matrix gene segment of avian influenza (H5N1) viruses from Bangladesh compared with other viruses. Green shading indicates viruses from Bangladesh sequenced and characterized in this study; yellow shading indicates previously described subtype H5N1/H9N2 reassortant influenza viruses (*1*,*2*) or those from GenBank. Numbers at the nodes represent bootstrap values.

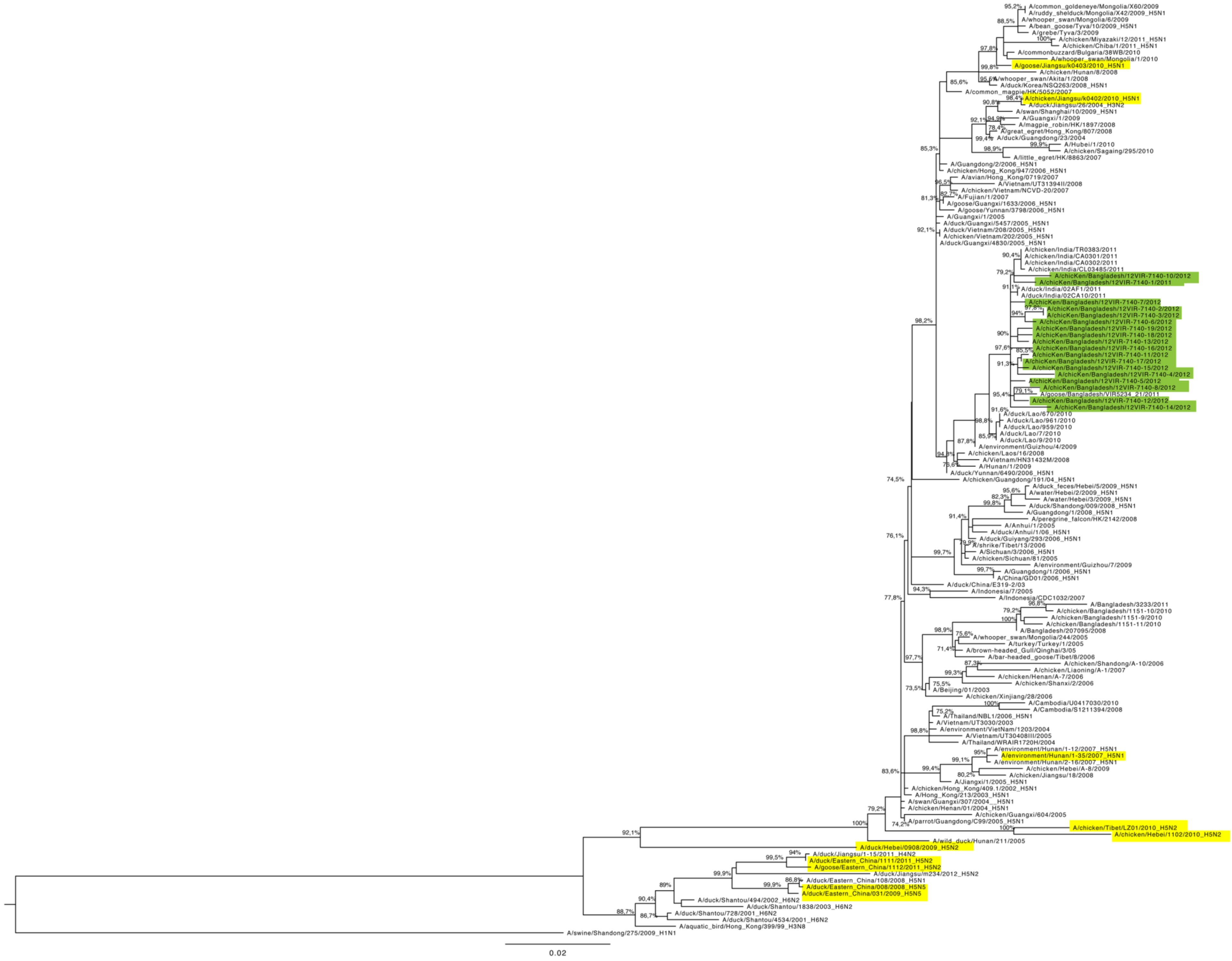

Technical Appendix Figure 6. Maximum-likelihood phylogenetic tree for the nucleoprotein gene segment of avian influenza (H5N1) viruses from Bangladesh compared with other viruses. Green shading indicates viruses from Bangladesh sequenced and characterized in this study; yellow shading indicates previously described subtype H5N1/H9N2 reassortant influenza viruses (*1*,*2*) or those from GenBank. Numbers at the nodes represent bootstrap values.

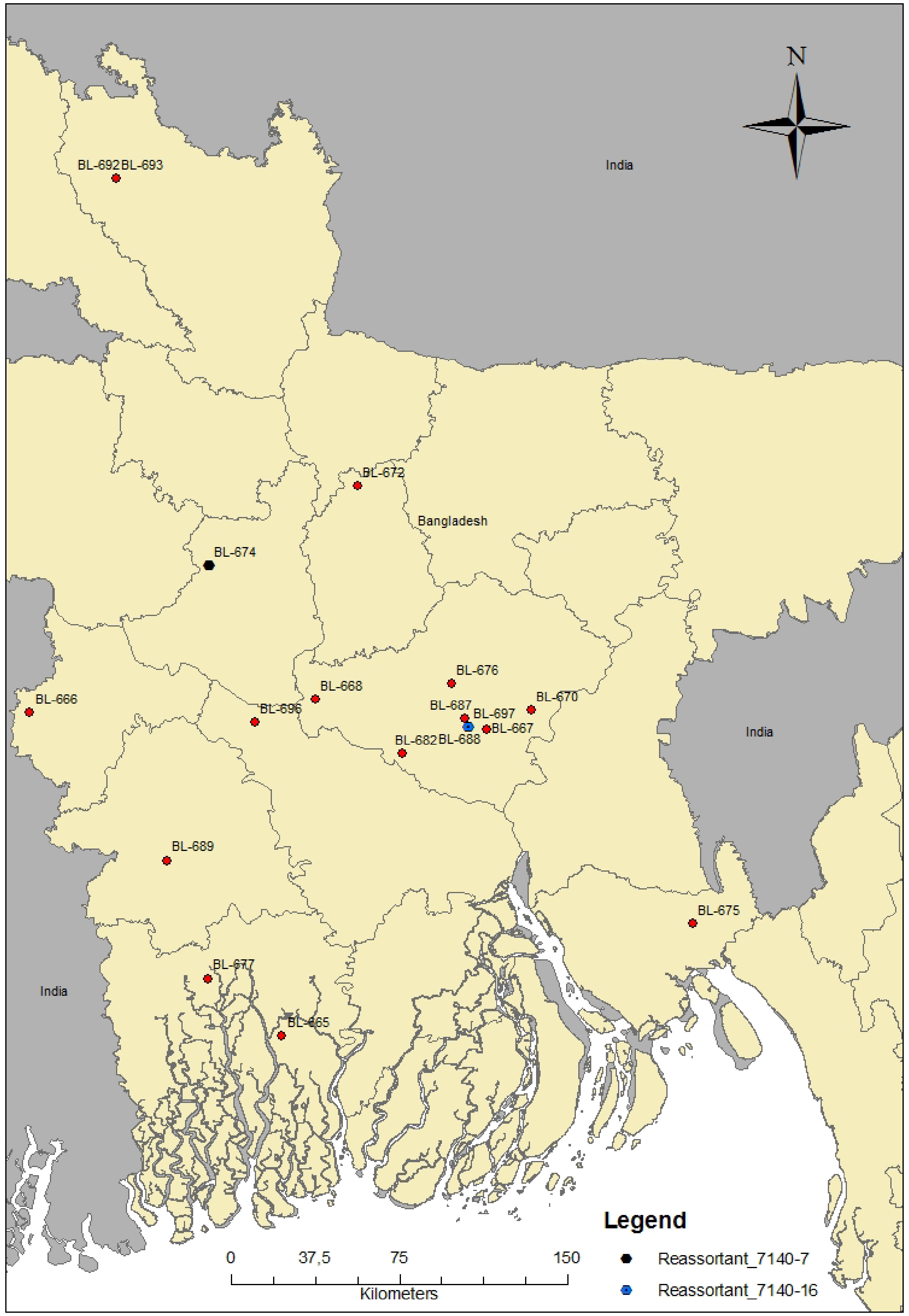

Technical Appendix Figure 7. Map of sample locations. Red dots indicate sample locations. The 2 reassortants are shown in different colors (IDs indicated in the map's legend). Map generated using ArcGIS 9.2 (ESRI, Redlands, CA, USA). The numbers at each bullet refer to the sample codes: 665 = 12VIR-7140-1/2011; 666 = 12VIR-7140-2/2011; 667 = 12VIR-7140-3/2011; 668 = 12VIR-7140-4/2011; 670 = 12VIR-7140-5/2011; 672 =12VIR-7140-6/2011; 674 =12VIR-7140-7/2011; 675 = 12VIR-7140-8/2011; 676 = 12VIR-7140-9/2011; 677 =12VIR-7140-10/2011; 682 =12VIR-7140-11/2011; 689 =12VIR-7140-12/2011; 692 =12VIR-7140-13/2011; 693 =12VIR-7140-14/2011; 696 =12VIR-7140-15/2011; 679 =12VIR-7140-16/2011; 686 =12VIR-7140-17/2011; 687 =12VIR-7140-18/2011; 688 =12VIR-7140-19/2011.

References

1. Zhao G, Gu X, Lu X, Pan J, Duan Z, Zhao K, et al. Novel reassortant highly pathogenic H5N2 avian influenza viruses in poultry in China. PLoS ONE. 2012;7:e46183. PubMed http://dx.doi.org/10.1371/journal.pone.0046183

2. Zhao S, Suo L, Jin M. Genetic characterization of a novel recombinant H5N2 avian influenza virus isolated from chickens in Tibet. J Virol. 2012;86:13836–7. PubMed http://dx.doi.org/10.1128/JVI.02595-12
